# Whole-Genome Sequence of *Streptococcus agalactiae* Strain S13, Isolated from a Fish Eye from a Nile Tilapia Farm in Southern Brazil

**DOI:** 10.1128/genomeA.00917-17

**Published:** 2017-08-31

**Authors:** César T. Facimoto, Roberta T. Chideroli, Daniela D. Gonçalves, Anderson O. do Carmo, Evanguedes Kalaphotakis, Ulisses de Pádua Pereira

**Affiliations:** a Department of Preventive Veterinary Medicine, Laboratory of Bacteriology in Fish (LABBEP), Universidade Estadual de Londrina, Londrina, Brazil; b Department of Preventive Veterinary Medicine and Public Health, Universidade Paranaense, Umuarama, Brazil; c Institute of Biological Science (ICB), Universidade Federal de Minas Gerais, Belo Horizonte, Brazil

## Abstract

*Streptococcus agalactiae* is an important pathogen to world aquaculture due to its high mortality rates in fish farms and consequent economic losses. Our study presents the complete genome sequence of strain S13, isolated from a tilapia farm outbreak in southern Brazil.

## GENOME ANNOUNCEMENT

*Streptococcus agalactiae* is a major pathogen responsible for mortality in fish farms around the globe and also affects other species, such as humans, cattle, mice, and frogs (1, 2). In fish farms, *S. agalactiae* causes high mortality in waters with temperature above 26°C, and early clinical signs of disease are erratic swimming, gasping, lethargy, melanosis, and exophthalmia (3–5). Transmission of *S. agalactiae* is possible through direct contact among individuals in cohabitation or indirectly through immersion in contaminated water of culture systems. Moreover, this agent manifests high virulence in fish, even at low 50% lethal dose (LD_50_) concentrations (3, 6). Human and bovine *S. agalactiae* strains are potentially able to infect fish farms, causing classical signs of streptococcosis (7).

The S13 strain was isolated in April 2015 from a tilapia farm outbreak in the north of Paraná state by the Laboratory of Fish Bacteriology (LABBEP). An ocular swab was collected aseptically from an individual with classical clinical signs of streptococcosis, streaked onto 5% sheep blood agar, and incubated at 28°C for 48 h. Later, colonies were diluted in 0.9 ml of Milli-Q water and centrifuged at 10,000 rpm for 10 min, followed by a phenol-chloroform-isoamyl alcohol protocol for DNA extraction (8). Sequencing of strain S13 was performed using an Illumina MiSeq platform (Illumina, Inc., San Diego, CA, USA), producing 2,389,562 reads that were imported to the CLC Genomics Workbench 8 software (Qiagen, USA). All reads with average Phred scores below 30, with the presence of ambiguities, and/or that were smaller than 50 bp in size were trimmed in the quality analysis step. Also, 10 nucleotides of every 3′ end of total reads were discarded.

After trimming, 2,317,875 reads were submitted to assembly using a *de novo* approach for the CLC Genomics Workbench software. A total of 23 contigs were generated, with coverage depth of 160× and an *N*_50_ value of 258,369, maximum length of 397,321 bp, and minimum length of 1,279 bp.

The reference genomes of strains SA-20-06 (GenBank accession no. NC_019048) and S25 (accession no. CP015976) were chosen due to their high identity scores with S13 by nucleotide BLAST. In order to organize the 23 contigs in a meaningful sequence, we used the CONTIGuator version 2 (9) software to align the contigs with the reference strains. The remaining gaps were filled using CLC Genomics Workbench 8 (Qiagen, USA), with subsequent rounds of short reads mapped against the scaffold (10), and gaps were filled using the overlapped reads layered in the intervals between contigs. Annotation of the genome was performed using the NCBI Prokaryotic Genome Annotation Pipeline (11).

The finished genome consists of a circular chromosome 1,835,156 bp in length, a 35.43% G+C content, 15 rRNA genes, 59 tRNA genes, and 182 pseudogenes. Multilocus sequence type (MLST) analysis was performed through the *Streptococcus agalactiae* MLST website (12), classifying the S13 strain as sequence type 552 (ST-552). The completed *S. agalactiae* S13 strain genome will help our understanding of the epidemiological dynamics and pathogenicity of this agent.

### Accession number(s).

The whole-genome sequence of the S13 strain has been deposited in the DDBJ/EMBL/GenBank public databases under accession number CP018623 and BioProject number PRJNA356737. The version described in this paper is the first version.
